# Identification of serum inflammatory markers as classifiers of lung cancer mortality for stage I adenocarcinoma

**DOI:** 10.18632/oncotarget.16784

**Published:** 2017-04-03

**Authors:** Claire L. Meaney, Adriana Zingone, Derek Brown, Yunkai Yu, Liang Cao, Bríd M. Ryan

**Affiliations:** ^1^ Laboratory of Human Carcinogenesis, Center for Cancer Research, National Cancer Institute, National Institutes of Health, Bethesda, MD, 20892, USA; ^2^ Genetics Branch, Center for Cancer Research, National Cancer Institute, National Institutes of Health, Bethesda, MD, 20892, USA

**Keywords:** lung cancer, biomarkers, survival, inflammation, IL-6

## Abstract

**Background:**

Lung cancer is the leading cause of cancer-related mortality worldwide. Low-dose CT (LDCT) imaging is now recommended to screen high-risk lung cancer individuals in the USA. LDCT has resulted in increased detection of stage I lung cancer for which the current standard of care is surgery alone. However, approximately 30% of these patients develop recurrence and therefore are in need of further treatment upon diagnosis. This study aims to explore blood-based inflammatory biomarkers to identify patients at high-risk of mortality for which additional treatment modalities can be offered at time of diagnosis.

**Patients and Methods:**

Recent work on a small panel of circulating cytokines identified elevated levels of IL-6, a pro-inflammatory cytokine, as an indicator of poor survival for lung cancer patients. To reflect the broader role of inflammation in lung cancer, we examined a large panel of 33 inflammatory proteins in the sera of 129 lung cancer patients selected from the National Cancer Institute-Maryland case-control study. To reduce heterogeneity, we specifically focused our study on stage I lung adenocarcinoma patients.

**Results:**

We replicated the previous observations that IL-6 is associated with prognosis of lung cancer and extended its utility to prognosis in this highly-selected population of stage I lung adenocarcinoma patients. In addition, we developed a multi-marker, combined prognostic classifier that includes the pro-inflammatory Th-17 cell effector cytokine, IL-17. Patients with high levels of IL-6 and IL-17A had a significantly adverse survival compared with patients with low levels (*P* for trend <0.0001). Patients in the high risk group, with high levels of both proteins had a 5-year survival rate of 46% in comparison to 93% for those with low levels of both markers. Furthermore, we validated the same trends for the IL-6 and IL-17A prognostic signature in an independent data set.

**Conclusions:**

The results identified here justify further investigation of this novel, combined cytokine prognostic classifier for the identification of high-risk stage I lung adenocarcinoma patients. This classifier has the much-needed potential to identify patients at high risk of recurrence and thus prospectively identify the subset of patients requiring more aggressive treatment regimens at the time of diagnosis.

## INTRODUCTION

Lung cancer is the leading cause of cancer-related mortality worldwide [1, 2]. Findings from the National Lung Screening Trial (NLST) indicated that the use of low dose helical CT (LDCT) as an annual screening tool reduced lung cancer related mortality by 20% in high risk smokers [3]. Annual LDCT screening is now recommended by the US Preventative Services Task Force for all individuals between the ages of 55 and 80 years with a history of smoking greater than 30 pack-years and those who have quit within 15 years [4]. The introduction of these screening recommendations has resulted in increased detection of stage I lung cancer. The current standard of care for these patients is surgery alone. However, between 20% and 30% of these patients will develop recurrence and are therefore in need of further treatment upon diagnosis [5, 6]. Thus, there is a need for the development of biomarkers to distinguish stage I patients in need of more aggressive treatment regimens at the time of diagnosis.

Cancer-related inflammation affects many aspects of cancer initiation and progression, including proliferation, survival, angiogenesis, and tumor metastasis [7–10]. The inflammatory cells that contribute to this phenotype are multi-faceted and include macrophages, neutrophils, and T cells, among others [11]. In addition, there is evidence that malignant epithelial cells can also secrete inflammatory cytokines [12]. Soluble immune factors such as cytokines and chemokines can be detected in patient sera, which, combined with the role of inflammatory proteins in lung cancer progression, could be leveraged as blood-based biomarkers of prognosis.

Recent work on a small panel of circulating cytokines identified elevated levels of IL-6, a pro-inflammatory cytokine, as an indicator of poor survival in lung cancer patients [13–24]. These studies included late stage or multiple stage cancers, as well as mixed histological subtypes—each of which can limit the refinement of a prognostic classifier. Moreover, most studies included a limited panel of immune markers. Given the complexity of the immune response in lung cancer and the multitude of cell types involved, we reasoned that examining a broad panel of inflammatory markers—including cytokines, chemokines, angiogenic and other pro-inflammatory factors—might identify a prognostic signature for survival. In addition, by limiting our analysis to a highly selected subset of patients, i.e., stage I lung adenocarcinoma, one would have a greater ability to identify a more refined prognostic classifier for the 20-30% of stage I patients at high-risk of recurrence.

## RESULTS

### Patient characteristics

The demographics of the population studied, in addition to clinical features relevant to this analysis, are shown in Table 1. The gender distribution among the population was close to proportionate (52% male and 48% female). The majority of patients in this population were stage IA (74%). Forty-six percent of patients were current smokers, while 33% were former smokers—never smokers comprised of 10% of the population included for this study. The median follow-up time was 47 months.

**Table 1:** Characteristics of NCI-MD participants included in our study

Characteristic	*N*	n	%
**Number of Subjects**	129		
**Age, years mean (range)**		65 (43-89)	
**Gender**			
Male		66	51%
Female		63	49%
**Race**			
African American		27	21%
European American		102	79%
**Stage**			
1a		95	74%
1b		34	26%
**Smoking Status**			
Never		13	10%
Former		47	37%
Current		69	53%
**Pack-years, mean (SD)**		40.1 (29.0)	
Light smokers (<10 pack-years)		19	15%
Moderate smokers (10-29 pack-years)		27	21%
Heavy smokers (≥30 pack-years)		83	64%
**Vital Status**			
Alive		83	64%
Deceased:			
Lung Cancer Specific		35	27%
Non-Lung Cancer Specific		11	9%

### Quality control metrics for the inflammatory marker detection levels

For each marker, we determined the number of samples that fell within the standard curve (Supplementary Table 1). The quality control criteria applied are detailed in the methods section and further details in Supplementary Table 2. Assays were flagged if the signal levels were above or below the curve fit range or below the detection range. Inflammatory markers for which 80% of samples were within the range of quantification were considered satisfactory for inclusion within the analysis (25 of the 33 markers analyzed). Samples that did not meet this detection criterion were excluded from the analysis—including IL-1α, IL-1β, IL-2, IL-4, IL-5, IL-13, GM-CSF and IL-12p70 (depicted in italics in Supplementary Table 1).

### Increased serum levels of inflammatory markers are associated with shorter survival

Kaplan Meier survival plots were generated for each marker and survival estimates for the ‘high’ (> median) and ‘low’ (≤ median) categories were compared. As explained in the methods section, a threshold-finding phase was conducted to examine quartile and median cut-points and determine the optimal cut-off value for the survival association analyses (Supplementary Table 3). The median cut-off value was chosen as the best stratification point that demonstrated best separation of prognosis within the survival estimates plots. To determine statistical differences between the survival characteristics of the two categories (above and below median), log-rank and univariable cox regression analyses were conducted. Five inflammatory markers of interest were identified based on their association with survival (Table 2). The Kaplan Meier plots, illustrating survival estimates of high and low levels of each marker are shown in Supplementary Figure 1. Elevated levels of IL-6, IL-17A, and Eotaxin-3 were associated with poor outcome (*P=0.012, P=0.049*, and *P=0.043*, respectively) (Table 2). Although the survival characteristics for CRP and IL-12p40 expression levels were not deemed statistically significant using the log-rank test (*P=0.061 and P=0.066* respectively), the relationships approached statistical significance (Supplementary Figure 1) (Table 2). Multivariable Cox regression modeling was used to determine whether the association between these five proteins with outcome was independent of potential confounders, including age, gender, race, stage (IA and IB), and smoking status (Table 2): IL-6 (HR, 2.34; 95% CI, 1.14-4.79) and IL-17A (HR, 2.10; 95% CI, 1.02-4.32) were independently associated with outcome, CRP (HR, 1.81; 95% CI, 0.90-3.65); was of borderline significance after adjustment for race, which is possibly a reflection of the differential expression of CRP by race [25].

**Table 2:** Summary of circulating inflammatory markers associated with survival of stage I adenocarcinoma patients

Variable	Log Rank	Univariable			Multivariable*		
		HR	95% CI	*P*	HR*	95% CI*	P*
**IL-6**							
≤ Median		1	Reference		1	Reference	
> Median	***0.012***	**2.38**	**1.19-4.78**	***0.014***	**2.34**	**1.14-4.79**	***0.020***
**CRP**							
≤ Median		1	Reference		1	Reference	
> Median	*0.061*	1.89	0.96-3.73	*0.066*	1.81	0.90-3.65	*0.098*
**IL-17A**							
≤ Median		1	Reference		1	Reference	
> Median	***0.049***	**1.99**	**0.99-4.00**	***0.053***	**2.10**	**1.02-4.32**	***0.044***
**IL-12p40**							
≤ Median		1	Reference		1	Reference	
> Median	*0.066*	1.88	0.95-3.71	*0.070*	1.77	0.86-3.61	*0.119*
**Eotaxin-3**							
≤ Median		1	Reference		1	Reference	
> Median	***0.043***	**2.03**	**1.01-4.08**	***0.048***	1.80	0.83-3.91	*0.137*

Abbreviations: HR, Hazard Ratio; CI, Confidence Interval.

*Adjusted for age, gender, stage (1a & 1b), race, smoking (never, former, current); bold text indicates statistical significance *P*<0.05.

Median values for each cytokine (pg/ml): IL-6 = 1.74; CRP = 3,824,875; IL-17A = 1.37;

IL-12p40 = 108.78; Eotaxin-3 = 13.38.

### Building a combined inflammatory marker classifier

We hypothesized that profiling a broad panel of inflammatory proteins would capture the multi-faceted role of inflammation in lung cancer progression. In addition, the use of multiple-marker classifiers has the potential to reduce misclassification. We first determined which cytokines were statistically correlated before determining statistical independence. As shown in Supplementary Table 4, significant positive correlations were observed between IL-6 and CRP (Rho=0.48, *P*<0.001) and IL-17A and IL-12p40 (Rho=0.37, *P*<0.001). Although these correlations are significant, all correlation coefficients were less than 0.5 and therefore considered as weak associations. To determine the statistical independence of each cytokine survival association, the multivariable models were additionally adjusted for the other cytokines of interest (Supplementary Table 5). After comparison of hazard ratio effect size, correlations and statistical independence of other markers, it was decided to employ IL-6, CRP and IL-17A within the combined classifier models going forward. Patients with elevated expression levels of these markers resulted in at least a 1.8 times greater chance of poor survival when compared to patients with low levels of the same marker.

**Figure 1:**
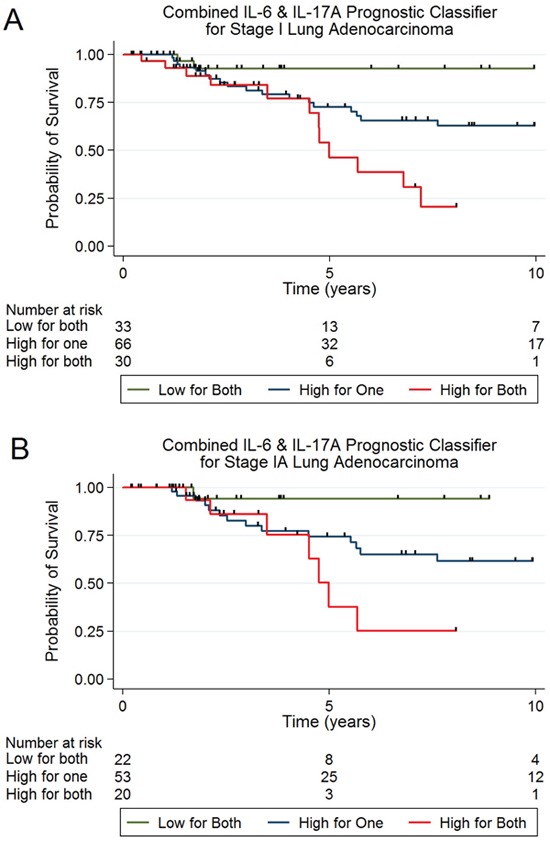
**(A)** Kaplan Meier plot illustrating survival estimates for stage IA & IB lung adenocarcinoma corresponding to categories of high and low IL-6 and IL-17A cytokine levels in a combined classifier. **(B)** Kaplan Meier plot illustrating survival estimates for stage IA lung adenocarcinoma corresponding to categories of high and low IL-6 IL-17A inflammatory protein levels.

The information criterion of each regression model was used to rank competing classifiers based on the goodness of fit and the number of parameters used within the model. Differences in information criterion values can be used to interpret the strength of evidence for one model versus another. Specifically, Akaike Information Criterion (AIC) was used as a measure of the relative quality of the models analyzed. AIC values for each (confounder adjusted) model were computed and ranked as shown in Supplementary Table 6, including various combinations of IL-6, CRP, and IL-17A. The combined classifier of IL-6 and IL-17A was ranked as the best model (AIC=269) to predict survival outcome of stage I lung adenocarcinoma patients. Patients with high levels of IL-6 and IL-17A had a significantly adverse survival compared with patients that had low levels (HR, 10.67; 95% CI, 2.17-52.44, *P*=0.004) (Table 3) (Figure 1A). Patients with high levels of both markers having a 5-year survival rate of 46% compared with 93% in patients with low levels of all three markers (Figure 1). The association with outcome for those with high expression for one of the two markers was also significant, yet the association was not as strong (Table 3). The second competing model, IL-6, IL-17A and CRP was ranked very closely (AIC=270). This three-marker classifier identified patients with high levels of all three markers as having a strong, significant association with poor survival (HR, 15.50; 95% CI, 1.81-132.57, *P=0.012*) (Table 4) and a 5-year survival rate of just 38% (Supplementary Figure 2A). All classifier models were adjusted for potential confounding variables including age, gender, self-reported race, stage (1a and 1b), smoking status (never, former, current), pack-years and sample collection date. Sample collection date refers to whether the blood sample was taken before surgery (negative number of days reflecting days prior to surgery) or after surgery (days after surgery given positive values).

Table 3The association of a combined IL-6 & IL-17A prognostic classifier with stage I lung adenocarcinoma survival

**Table:** 

Classifier: IL-6 & IL-17A (Stage IA and IB)	N	%	Univariable			Multivariable*		
			HR	95% C.I.	*P*	HR*	95% C.I.*	P*
Low for both	33	25.6	1	Reference		1	Reference	
High for one	66	51.1	4.59	1.08-19.59	*0.039*	5.13	1.14-23.25	***0.034***
High for both	30	23.3	9.64	2.13-43.58	***0.003***	10.67	2.17-52.44	***0.004***

*P* trend: 0.001.

* adjusted for age, gender, stage (1a & 1b), race, smoking (never, former, current), pack-years, and sample collection date.

**Table:** 

Classifier: IL-6 & IL-17A ((Stage IA only)	N	%	Univariable			Multivariable*		
			HR	95% C.I.	*P*	HR*	95% C.I.*	P*
Low for both	22	23.2	1	Reference		1	Reference	
High for one	53	55.8	5.57	0.74-42.16	*0.097*	3.86	0.49-30.70	***0.202***
High for both	20	21.0	11.01	1.34-90.22	***0.025***	7.81	0.83-73.07	***0.072***

*P* trend: 0.051.

* adjusted for age, gender, race, smoking (never, former, current), pack-years, and sample collection date.

Table 4The association of a combined IL-6, CRP & IL-17A prognostic classifier with stage I lung adenocarcinoma survival

**Table:** 

Classifier: IL-6, CRP & IL-17A (Stage IA and IB)	N	%	Univariable			Multivariable*		
			HR	95% C.I.	*P*	HR*	95% C.I.*	P*
Low for all three	24	18.6	1	Reference		1	Reference	
High for one or two	81	62.8	6.42	0.87-47.49	*0.069*	7.66	0.99-59.55	*0.052*
High for all three	24	18.6	15.17	1.92-119.97	***0.010***	15.50	1.81-132.57	***0.012***

*P* trend: 0.003.

* adjusted for age, gender, stage (1a & 1b), race, smoking (never, former, current), pack-years and sample collection date.

**Table:** 

Classifier: IL-6, CRP & IL-17A (Stage IA only)	N	%	Univariable			Multivariable*		
			HR	95% C.I.	*P*	HR*	95% C.I.*	P*
Low for all three	16	16.8	1	Reference		1	Reference	
High for one or two	63	66.3	3.16	0.42-24.02	*0.265*	2.06	0.26-16.16	*0.493*
High for all three	16	16.8	9.31	1.14-76.16	***0.038***	6.44	0.69-59.73	***0.101***

*P* trend: 0.052.

* adjusted for age, gender, race, smoking (never, former, current), pack-years and sample collection date.

As mentioned, results from the NLST show that annual screens of high-risk individuals with LDCT reduces lung cancer mortality and diagnoses a predominance of early-stage lung cancer, particularly stage IA. In our cohort, despite being diagnosed with early stage disease, 23/95 (24%) of patients died. We therefore investigated whether the association with survival of the IL-6 plus IL-17A classifier and the IL-6, CRP and IL-17A classifier was specifically associated with survival in stage IA lung cancer patients. As shown in Table 3 and Figure 1B, the association of the IL-6 and IL-17A classifier with survival for stage IA (n=95) was border-line significant after adjustment for all relevant variables (HR, 7.81; 95% CI, 0.83-73.07, *P*=0.072) with a *p*_trend_ of 0.051; therefore, this classifier was able to identify a sub-population of high-risk stage IA patients. The association of the IL-6, CRP and IL-17A classifier with survival for stage IA (n=95) was also demonstrated a border line significant *p*_trend_ of 0.052 (Table 4 and Supplementary Figure 2B).

There were 11 patients in our study that died of causes other than lung cancer. We therefore conducted a competing risks analysis to address this potential factor and found that the association between the IL-6 and IL-17A classifier and survival was not affected by censoring of the 11 individuals who died of causes other than lung cancer (fully adjusted model, for patients with high levels of both markers (HR: 11.57; 95% CI, 2.132.10 – 63.0662.13, *P*=0.005).

To confirm the findings of this discovery analysis 113 independent, stage I, NSCLC, adenocarcinoma samples from the NCI-MD case control study were collected and analyzed as a validation cohort. Details of the population characteristics of the validation cohort are provided in Supplementary Table 7. The same quality control metrics for the inflammatory marker detection levels were applied to the validation cohort. Protein expression levels were analyzed using the same methods as for the discovery data set and the same statistical analyses were conducted to determine whether the IL-6 plus IL-17A classifier findings from the original dataset held true for this independent sample set. Sample detection levels were within the defined measurement criteria. Five control samples were used to determine inter-plate variation—the coefficients of variance for IL-6 and IL-17A were 8.7% and 14.7% respectively (see Supplementary Table 8). Again, we found that patients with high expression levels of IL-6 and IL-17A were at higher risk of poor prognosis in comparison to those with low expression levels of both of these markers, *p*_trend_ *0.064* (Supplementary Table 9 and Supplementary Figure 3).

## DISCUSSION

In this study, we identified a combined, inflammatory-based prognostic classifier for stage I lung adenocarcinoma patients. The classifier leverages blood-based biomarkers of inflammation to identify sub groups of patients that are at a high risk of mortality, despite having been diagnosed with early stage disease. Patients with elevated serum levels of IL-6 and IL-17A have a strong association with poor outcome. These high-risk patients have a 5-year survival rate of 46%, considerably lower than the 93% 5-year survival rate of patients with low levels of IL-6 and IL-17A. The intermediate group, patients with high levels of one of the two markers, also had a significant association with survival and a 5-year survival rate of 73%. A similar trend was found in the validation study using independent stage I adenocarcinoma patients; patients with high circulating levels of both IL-6 and IL-17A had an increased risk of poor prognosis. The addition of CRP into the combined classifier may help identify those at high risk in comparison to those at intermediate risk, however this will have to be investigated further. The second competing model of IL-6, CRP and IL-17A also showed that patients with elevated serum levels of all three markers had strong association with poor outcome (5-year survival rate of 38%).

The inflammatory markers within this combined classifier have reported mechanistic roles in lung cancer. IL-6 is a multifunctional player in cancer progression—it is known for its role in pro-inflammatory immune responses, cell survival, apoptosis and proliferation [26]. IL-6 signals via the IL-6R/gp130 complex and triggers downstream effectors STAT3 and Ras [26, 27]. Reports have suggested that elevated levels of IL-6 are mechanistically linked to poor cancer prognosis via IL-6-induced activation of miR-21 via the activation of the STAT3 pathway [28]—miR-21 has been associated with poor survival in lung cancer in multiple studies [29, 30]. Although this analysis is based on circulating levels of biomarkers and therefore the origin of the signal is not established, IL-6 has been shown to be secreted by tumor infiltrating immune cells [26] and tumor epithelial cells in lung cancer [12]. Thus, IL-6 could be tumor derived, or indeed released from tumor associated M1 macrophages and neutrophils [11, 12]. Elevated levels of IL-6 have been previously linked to poor lung cancer survival [13–24, 31, 32]. However, these studies involved multiple histological subtypes and tumor stages. To our knowledge, ours is the first study to examine IL-6 with lung cancer prognosis in such a refined population.

IL-17A is a pro-inflammatory cytokine mainly produced by activated Th-17 cells and has been shown to play an active role in many cancers [33]. However, high levels of IL-17A expression, measured by immunohistochemistry in non-small-cell lung tumor samples, has been associated with poor lung cancer survival [34]. Additional evidence suggests that IL-17A drives EMT via STAT-3 signaling in lung cancer, perhaps a mechanism to explain the association with poor prognosis [35–37]. Interestingly, IL-17A can promote tumor growth through an IL-6/STAT-3 signaling pathway [38].

Although the IL-6 plus IL-17A model was ranked as the most robust model as defined by statistical information criteria, the competing model of IL-6, CRP and IL-17A ranked very closely and may help in identification of intermediate risk patients. CRP is a hallmark of acute systemic inflammation [39] and is a highly sensitive, yet unspecific, marker of inflammation. The addition of CRP to the model was of borderline significance when race was taken into account. This is likely reflective of the relationship between CRP levels and race and indicates that further refinement of this classifier in European American and/or African American populations alone is warranted. Although the reasons for elevated CRP levels in cancer patients is not clearly understood, other studies have also supported CRP as an indicator of poor outcome in non-small cell lung cancer [40–44]. Furthermore, CRP has been noted as a marker of lung cancer risk [32] and interestingly, as a predictor of response to anti-EGFR gefitinib therapy [44]. Although the main site of CRP production is the liver, additional evidence shows extra-hepatic expression of CRP, including in lung epithelial cells [45]. It isn't clear how CRP is related to lung cancer progression. However, given that its main function is in activating the complement system, it is possible that high levels of this protein create a permissive inflammatory state characterized by increased oxidative and nitrosative stress.

Our findings build upon previous findings pertaining to serum based classifiers of lung cancer and lung cancer prognosis. Although we previously identified an association between IL-8 and lung cancer prognosis [13], we did not find evidence of a relationship between IL-8 with prognosis in this study. This is possibly because this study included only stage I lung adenocarcinoma patients, while our previous study included multiple histological subtypes.

Our study has several strengths and limitations. The inclusion of a homogenous cohort of stage I only and lung adenocarcinoma only patients strengthened our ability to identify a refined prognostic classifier. Also, our selection of this population was driven by the increase in such diagnoses in the era of LDCT screening. We previously found that cytokine profiles differ between European Americans and African Americans; however, due to the limited number of African Americans enrolled in this study, we were not able to perform a stratified analysis to examine whether race-specific differences exist in regards to this classifier. However, studies of individual racial groups in the future are warranted. Similarly, studies disagree regarding the utility of systemic chemotherapy for stage IB lung cancer patients [46–50]. We attempted to assess the ability of the classifier to predict outcome in this sub-group of patients and the model demonstrated similar trends to those observed in stage IA, however, the stratified analysis was limited by small sample size (n=34). The majority of patients within this study population (64%) were defined as heavy smokers (>30 pack-years) and 45% of the total population analyzed fall within the NLST screening criteria. We tested the classifier on a restricted population of NLST-eligible patients alone (N=58) and found that the same trend for the IL-6-IL-17A classifier. Another potential limitation of this study was the application of medians as the defined cut-off. Although the median cut-off was determined as the best stratification threshold within this analysis—further studies will be needed to refine the most accurate cut-off of high and low biomarker criteria. Encouragingly however, using the median levels of IL-6 as defined in previous studies [13, 32], i.e., 2.1 pg/ml, we again found an increased hazard of death associated with increased levels of IL-6 (data not shown). This study presents a discovery analysis, and confirmatory validation study, that indicate patients with high levels of circulating IL-6 and IL-17A are at risk of poor prognosis in comparison to those with low levels of both markers.

The combined prognostic role of IL-6, CRP plus IL-17A in stage I lung adenocarcinoma has not been reported previously. The significance of IL-17A, CRP and IL-6 suggests that not just Th-2 or Th-17 cells are important in lung cancer progression and supports our hypothesis that the inclusion of a broader cytokine panel can capture the multi-faceted role of inflammation in lung cancer. Currently, IL-6 targeted therapies are in development [26, 51–53] as are anti-miR-21 treatment strategies. Recently, IL-6 blockade was shown to inhibit mutant *KRAS* driven lung cancer [53], indeed several studies have shown increased IL-6 expression in lung tumors with mutant *KRAS* [54]—evidence of this relationship can also be observed from a parallel study on-going in our group (Supplementary Figure 4). Moreover, IL-17A production by Th17 cells induces more production of IL-6 which, in turn, activates STAT3, upregulating pro-survival proangiogenic genes [38, 54]. While our study has specifically assessed this classifier in terms of prognosis, it is possible that IL-6-based classifiers could also be used for predicting response to IL-6-targeted therapies, or indeed, response to first line immunotherapy. These possibilities will require further research and investigation.

## MATERIALS AND METHODS

### Study population

The patient samples selected for this nested case study were selected from the National Cancer Institute-Maryland (NCI-MD) lung cancer study. Patients were radiotherapy/chemotherapy treatment-naïve, stage I, lung adenocarcinoma patients. Patient samples that met these criteria were chosen based on availability of a serum biospecimen. The study population accrual and eligibility criteria for the case-control study were previously described [16, 32]. Briefly, all cases had histologically confirmed non-small cell lung cancer. Participants were excluded if they had a known diagnosis of HIV, hepatitis C or hepatitis B virus. All participants were self-reported African Americans or European American residing in Metropolitan Baltimore area or the Maryland Eastern Shore. All participants completed an interviewer-administered questionnaire that covers lifestyle, medical, and demographic information. Never smokers were defined as those who had smoked fewer than 100 cigarettes in their entire lifetime. Former smokers were defined as individuals who had quit smoking for at least 1 year prior to interview. This nested case-case study included serum samples from 129 stage I lung adenocarcinoma patients. Blood was taken at the time of interview/diagnosis. Institutional Review Board approval has been obtained from all participating institutes and the National Institutes of Health. Detailed information regarding the 129 patients is outlined in Table 1.

### Mortality and survival determination

To obtain data on lung cancer-specific mortality, the date and cause of death were obtained from the National Death Index which provides cause of death codes. The linkage process has been previously described [16]. Lung cancer-specific death was defined as a case with lung cancer listed as the primary, secondary or tertiary cause of death or death due to another cancer within 2 years of the lung cancer diagnosis. TNM staging was re-classified using AJCC 7^th^ edition. Survival time was calculated from date of surgery to date of either last known follow-up (last National Death Index update on 12/31/2012) or date of death due to lung cancer.

### Inflammatory marker measurement

Concentrations of 33 inflammatory markers were measured on serum samples of 129 cases using a highly sensitive and analytically validated electrochemiluminescence VPLEX immunoassay (MSD® Rockville, MD) at the Frederick National Laboratory for Cancer Research, following the manufacturer's instructions. Serum samples from all participants were randomly distributed across the plates, control samples to assess inter-plate variability, and analyte-specific standards to generate standard curves were also included with each plate. The total panel of 33 inflammatory markers analyzed is detailed in Supplementary Table 10. Briefly, 25μl of patient sera were assayed, following the manufacturer's protocol, for circulating levels of each inflammatory marker of interest using the MSD® V-PLEX Chemokine Panel 1 Kit (Eotaxin, Eotaxin-3, IL-8, IP-10, MCP-1, MCP-4, MDC, MIP-1α, MIP-1β, TARC), V-PLEX Pro-inflammatory Panel 1 Kit (IFN-γ, IL-10, IL-12p70, IL-13, IL-1β, IL-2, IL-4, IL-6, TNF-α), V-PLEX Cytokine Panel 1 Kit (GM-CSF, IL-1α, IL-5, IL-7, IL-12p40, IL-15, IL-16, IL-17A, TNF-β, and VEGF-A). Samples were further analyzed for the concentrations of CRP, SAA, sICAM-1 and sVCAM-1. All signal results were extrapolated into concentrations (pg/ml) from the standard curves. Two control samples (random serum samples from healthy volunteers) were also included on each plate to assess intraplate variance (see Supplementary Table 11 for coefficients of variance). To ensure quality data for further analyses and interpretation, detection level criteria were applied to the measurements obtained for each marker (Supplementary Table 1) based on fit curve ranges defined by the plate-specific-standard curves generated for each analyte using standard dilutions (computations were conducted using Workbench 4.0 (MSD® Rockville, MD). All measurements that lay within the quantification range (Supplementary Table 2) were included within the analysis without further computation. Expression level values of less than the lower limit of detection (defined as the value 2.5. standard deviation above the background signal) were assigned a value of one half the lower limit of detection. The median value of each marker was chosen as the cut-off value to classify high and low levels of the protein. This decision was based on a threshold-finding analysis examining the survival associations of each marker expression levels when split into quartiles and using the median value as a cut-off value. Supplementary Table 3 presents the comparison of survival associations for five markers of interest using quartile and median cut-off values (these five markers were selected for presentation based on their strong associations with survival established from log rank tests and Kaplan Meier plots, discussed further in the results section).

### Statistical analysis

The association between inflammatory markers and survival was initially assessed using the method of Kaplan and Meier. Statistical differences in survival between the ‘high’ (> median) and ‘low’ (≤ median) categories were evaluated using Log-rank tests of equality. To test the magnitude of association between inflammatory markers and lung cancer-specific survival, hazard ratios (HR) were estimated using univariable and multivariable Cox proportional hazards regression modeling. Multivariable analyses were adjusted to control for the following potential confounding variables: age (continuous), gender (male/female), race (African American/European American), current smoking status (never/former/current), pack-years (continuous), sample collection date (a continuous variable measured in days prior to and after surgery) and stage (stage IA, stage IB). Variables selected for adjustment were based on standard prognostic variables used in the literature. Time from lung cancer diagnosis until death or date of last known follow up was used to estimate the survival timescale and failure was described as lung cancer-specific death. All statistical analyses were performed using STATA® 13.0 (StataCorp, College Station, TX). In this study, causes of death other than lung cancer were censored (n=11). As competing risks are distinct from standard censoring, we performed a competing risks regression based on the method of Fine and Gray [55] using the stcrreg function in STATA. A new variable was generated to specify the competing events (death from cancer and death from another cause).

## SUPPLEMENTARY MATERIALS FIGURES AND TABLES


