# Supplementary material for: Identification of serum inflammatory markers as classifiers of lung cancer mortality for stage I adenocarcinoma
Source: Oncotarget. 2017 Apr 3;8(25):40946–57. doi: 10.18632/oncotarget.16784 (PMC5522266; doi:10.18632/oncotarget.16784)
Supplement: Supplementary file 1 [file oncotarget-08-40946-s001.pdf]

Identification of serum inflammatory markers as classifiers of lung cancer mortality for stage I adenocarcinoma

SUPPLEMENTARY FIGURES AND TABLES

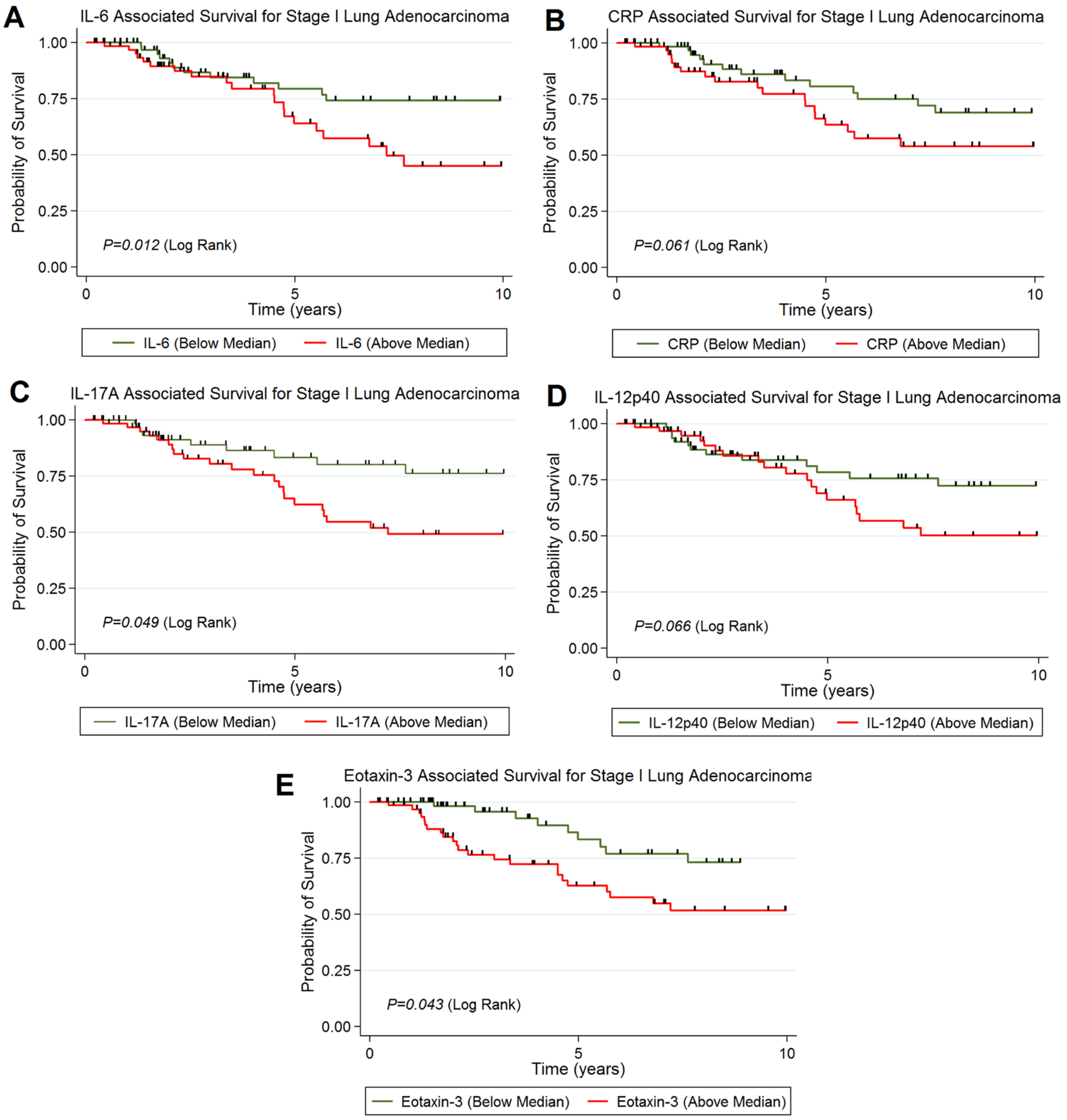

Supplementary Figure 1: Kaplan Meier plot illustrating survival estimates for stage I lung adenocarcinoma corresponding to levels of serum inflammatory markers detected upon diagnosis **(A)** IL-6, **(B)** CRP, **(C)** IL-17A, **(D)** IL-12p40 and **(E)** Eotaxin-3.

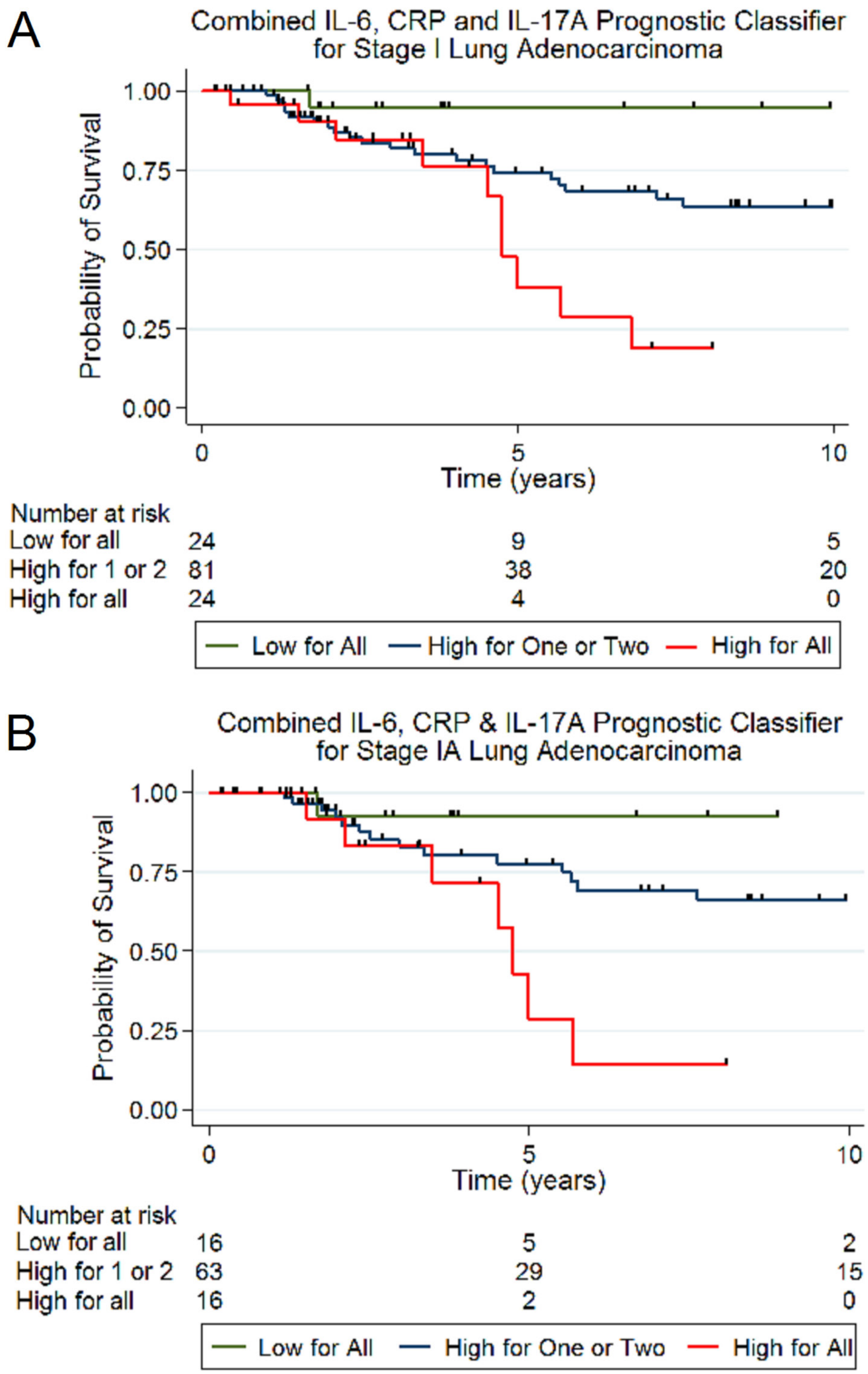

Supplementary Figure 2: **(A)** Kaplan Meier plot illustrating survival estimates for stage IA&B lung adenocarcinoma corresponding to categories of high and low IL-6, CRP, IL-17A inflammatory protein levels. **(B)** Kaplan Meier plot illustrating survival estimates for stage IA lung adenocarcinoma corresponding to categories of high and low IL-6, CRP, IL-17A inflammatory protein levels.

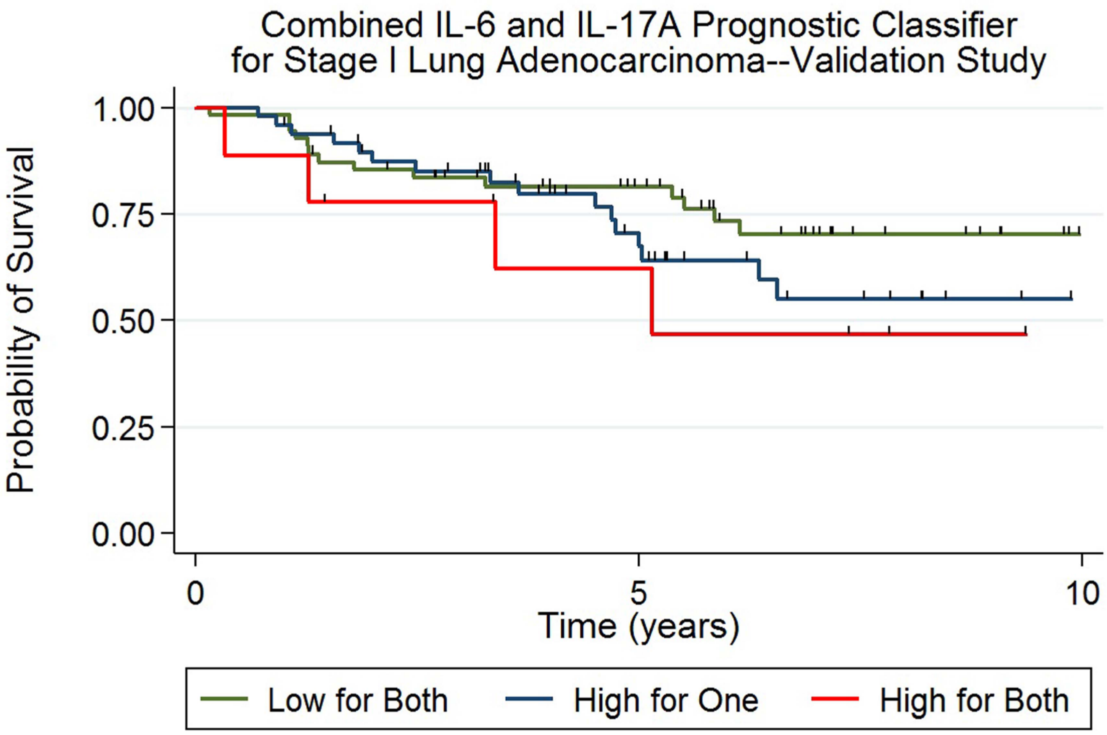

Supplementary Figure 3: Kaplan Meier plot illustrating survival estimates for Validation Study of stage IA&B lung adenocarcinoma corresponding to categories of high and low IL-6 and IL-17A inflammatory protein levels.

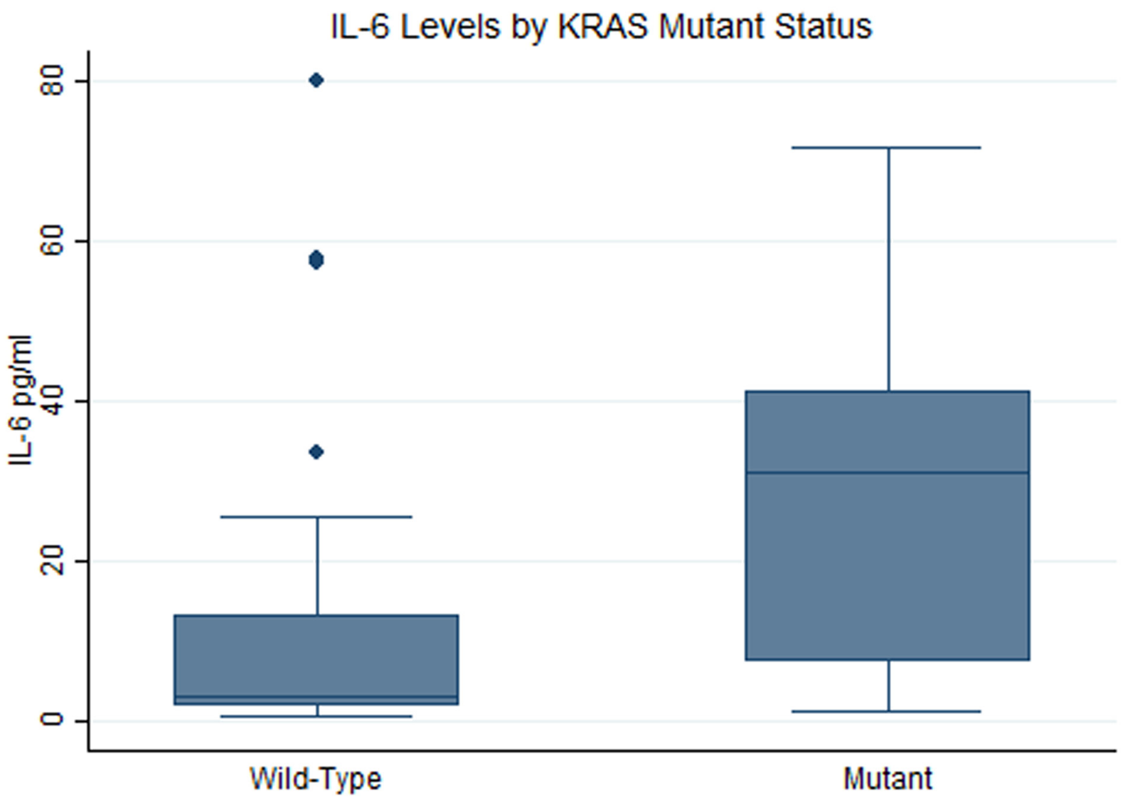

Supplementary Figure 4: Increased IL-6 levels with KRAS mutant status (p<0.05).

Supplementary Table 1: Distribution of analyte detection for each inflammatory marker

Assay	Above Fit Curve Range	Below Detection Range	Below Fit Curve Range	In Detection Range	% In Detection Range
**Eotaxin**	**0**	**0**	**0**	**129**	**100**
**IFN-γ**	**0**	**0**	**0**	**129**	**100**
**IL-12p40**	**0**	**0**	**0**	**129**	**100**
**IL-15**	**0**	**0**	**0**	**129**	**100**
**IL-16**	**0**	**0**	**0**	**129**	**100**
**IL-7**	**0**	**0**	**0**	**129**	**100**
**MCP-1**	**0**	**0**	**0**	**129**	**100**
**sICAM-1**	**0**	**0**	**0**	**129**	**100**
**sVCAM-1**	**0**	**0**	**0**	**129**	**100**
**TARC**	**0**	**0**	**0**	**129**	**100**
**VEGF**	**0**	**0**	**0**	**129**	**100**
**CRP**	**1**	**0**	**0**	**128**	**99.2**
**IL-6**	**0**	**1**	**0**	**128**	**99.2**
**IP-10**	**0**	**1**	**0**	**128**	**99.2**
**MCP-4**	**0**	**1**	**0**	**128**	**99.2**
**MDC**	**0**	**1**	**0**	**128**	**99.2**
**MIP-1α**	**0**	**1**	**0**	**128**	**99.2**
**MIP-1β**	**0**	**1**	**0**	**128**	**99.2**
**TNF-α**	**0**	**1**	**0**	**128**	**99.2**
**IL-10**	**0**	**3**	**0**	**126**	**97.7**
**IL-17A**	**0**	**6**	**0**	**123**	**95.4**
**TNF-β**	**0**	**5**	**3**	**121**	**93.8**
**SAA**	**8**	**0**	**0**	**120**	**93.0**
**IL-8**	**15**	**0**	**0**	**114**	**88.4**
**Eotaxin-3**	**0**	**21**	**1**	**107**	**83.0**
IL-2	*0*	*53*	*3*	*73*	*56.6*
IL-4	*0*	*48*	*12*	*69*	*53.5*
IL-5	*0*	*31*	*31*	*67*	*51.9*
IL-13	*0*	*57*	*8*	*64*	*49.6*
GM-CSF	*0*	*66*	*0*	*63*	*48.8*
IL-1β	*0*	*17*	*51*	*61*	*47.3*
IL-1α	*0*	*38*	*43*	*48*	*37.2*
IL-12p70	*0*	*83*	*11*	*35*	*27.1*

Italics indicate cytokine detection level below 80%.

Supplementary Table 2: Summary of detection ranges for each assay and each plate

Cytokine Panel		Pro-Inflammatory Panel	
**Analyte**	**Detection Range (pg/mL)**	**Analyte**	**Detection Range (pg/mL)**
GM-CSF	0.07 - 950	IFN-γ	0.16 - 1460
IL-12p40	0.23 - 3070	IL-10	0.02 - 334
IL-15	0.08 - 683	IL-12 p70	0.07 - 421
IL-16	0.98 - 2810	IL-13	0.44 - 466
IL-17A	0.15 - 5670	IL-1β	0.01 - 495
IL-1α	0.06 - 358	IL-2	0.05 - 1460
IL-5	0.04 - 817	IL-4	0.01 - 198
IL-7	0.07 - 628	IL-6	0.05 - 767
TNF-β	0.02 - 581	IL-8	0.03 - 495
VEGF	0.18 - 996	TNF-α	0.04 -312
**Chemokine Panel**		**Vascular Injury Panel**	
**Analyte**	**Detection Range (pg/mL)**	**Analyte**	**Detection Range (pg/mL)**
Eotaxin	1.35 - 1510	CRP	2.09 - 182500
Eotaxin-3	1.71 – 5020	SAA	23.94 - 207500
IP-10	0.05 – 2650	sICAM-1	1.59 - 45900
MCP-1	0.06 – 488	sVCAM-1	7.38 - 49150
MCP-4	1.09 – 645		
MDC	1.61 – 10100		
MIP-1α	0.75 – 1040		
MIP-1β	0.27 – 1050		
TARC	0.08 - 1470		

Supplementary Table 3: The associations between survival and levels of circulating inflammatory markers IL-6, CRP, IL-17A, IL-12p40 and Eotaxin-3 for stage I adenocarcinoma patients

Variable	Univariable			Multivariable*		
	HR	95% CI	*P*	HR	95% CI	*P*
IL-6						
First quartile	1	Reference		1	Reference	
Second quartile	1.76	0.59-5.27	0.310	1.79	0.60-5.39	0.300
Third quartile	2.34	0.83-6.63	0.109	2.04	0.70-5.94	0.193
Fourth quartile	**2.92**	**1.11-7.72**	**0.031**	**2.88**	**1.01-7.78**	**0.037**
p_{trend}	1.4	1.04-1.88	0.025	**1.39**	**1.02-1.89**	**0.037**
≤Median	1	Reference		1	Reference	
≥Median	**2.38**	**1.18-4.78**	**0.014**	**2.34**	**1.14-4.79**	**0.020**
CRP						
First quartile	1	Reference		1	Reference	
Second quartile	0.72	0.25-2.07	0.542	0.68	0.29-2.03	0.488
Third quartile	1.04	0.38-2.88	0.936	1	0.36-2.76	0.993
Fourth quartile	2.05	0.82-5.15	0.125	1.97	0.72-5.41	0.186
p_{trend}	1.35	0.98-1.86	0.063	1.32	0.94-1.86	0.109
≤Median	1	Reference		1	Reference	
≥Median	1.89	0.96-3.73	0.066	1.81	0.90-3.65	0.098
IL-17A						
First quartile	1	Reference		1	Reference	
Second quartile	0.82	0.27-2.56	0.735	1.09	0.33-3.65	0.886
Third quartile	1.91	0.73-5.03	0.191	2.33	0.82-6.57	0.11
Fourth quartile	1.46	0.53-4.02	0.465	1.83	0.64-5.24	0.263
p_{trend}	1.22	0.90-1.66	0.209	1.27	0.93-1.73	0.132
≤Median	1	Reference		1	Reference	
≥Median	1.99	0.99-4.00	0.053	**2.1**	**1.02-4.32**	**0.044**
IL-12p40						
First quartile	1	Reference		1	Reference	
Second quartile	0.61	0.21-1.75	0.354	0.66	0.23-1.95	0.454
Third quartile	1.18	0.44-3.16	0.739	1.21	0.44-3.32	0.716
Fourth quartile	1.73	0.71-4.19	0.226	1.72	0.69-4.33	0.250
p_{trend}	1.29	0.95-1.75	0.097	1.26	0.92-1.72	0.156
≤Median	1	Reference		1	Reference	
≥Median	1.88	0.95-3.71	0.070	1.77	0.86-3.61	0.119
Eotaxin-3						
First quartile	1	Reference		1	Reference	
Second quartile	0.93	0.29-2.95	0.903	0.92	0.28-2.99	0.890
Third quartile	1.32	0.44-3.94	0.622	1.11	0.36-3.64	0.336
Fourth quartile	2.70	0.97-7.51	0.056	2.32	0.77-7.01	0.136
p_{trend}	**1.48**	**1.06-2.07**	**0.020**	**1.41**	**0.98-2.02**	**0.062**
≤Median	1	Reference		1	Reference	
≥Median	**2.03**	**1.01-4.08**	**0.048**	1.8	0.83-3.91	0.137

(HR=Hazard Ratio, CI=Confidence Interval); *Adjusted for age, gender, stage (1a & 1b), race, smoking (never, former, current); Bold text indicates statistically significance p<0.05.

– These five markers were selected for presentation based on their strong associations with survival established from log rank tests and Kaplan Meier plots–.

Supplementary Table 4: Correlations between inflammatory markers

**Cytokine:**	**Spearman Rank Correlation Coefficient (ρ)**				
	**IL-6**	**CRP**	**IL-17**	**IL-12p40**	**Eotaxin-3**
**IL-6**		**0.48**	0.07	0.04	0.15
**CRP**			0.17	0.08	**0.25**
**IL-17A**				**0.37**	0.12
**IL-12p40**					0.07
**Eotaxin-3**					

Bold text indicated statistical significance *P<0.05*.

Supplementary Table 5: Testing the statistical independence of biomarker survival associations

Markers of Interest:			Further adjusted for:				
			**IL-6**	**CRP**	**IL-17A**	**IL-12p40**	**Eotaxin-3**
**IL-6**	HR*	2.34		2.30	2.55	2.35	2.58
	(95 % CI)	1.14-4.79		1.06-4.97	1.21-5.37	1.15-4.83	1.25-5.34
	*P**	*0.020*		***0.033***	***0.014***	***0.019***	***0.011***
**CRP**	HR*:	1.81	1.67		1.78	1.71	1.90
	(95 % CI)	0.90-3.65	0.80-3.49		0.88-3.62	0.85-3.44	0.94-3.83
	P	*0.098*	*0.175*		*0.108*	*0.134*	*0.074*
**IL-17A**	HR*:	2.10	2.22	2.02		1.90	2.00
	(95 % CI)	1.02-4.32	1.07-4.58	0.97-4.19		0.92-3.98	0.97-4.14
	P	***0.044***	***0.031***	*^0.060*		*^0.085*	*^0.062*
**IL-12p40**	HR*:	1.77	1.66	1.74	1.70		1.69
	(95 % CI)	0.86-3.61	0.80-3.46	0.85-3.57	0.82-3.53		0.83-3.44
	P	*0.119*	*0.176*	*0.128*	*0.156*		*0.152*
**Eotaxin-3**	HR*:	1.80	1.72	1.79	1.78	1.65	
	(95 % CI)	0.83-3.91	0.79-3.73	0.82-3.91	0.83-3.83	0.76-3.59	
	P	*0.137*	*0.171*	*0.143*	*0.141*	*0.204*	

* indicates baseline models were adjusted for age, gender, race, stage, smoking; bold text indicates statistical significance P<0.05; ^ indicates a change in statistical significance from baseline model; † indicates a change in effect ±10% of baseline hazard ratio.

Supplementary Table 6: Classifier model ranking using the akaike information criterion

Classifier	AIC*
IL-6	275
IL-17A	274
CRP	276
IL-6 & IL-17A	269
IL-6 & CRP	276
IL-17A & CRP	272
IL-6, CRP & IL-17A	270

*Model adjusted for age, gender, stage, race, smoking status, pack-years and sample collection date.

Supplementary Table 7: Characteristics of NCI-MD participants included in our validation study

**Characteristic**	***N***	**n**	**%**
**Number of Subjects**	113		
**Age, years mean (range)**		67 (41-87)	
**Gender**			
Male		65	57.5%
Female		48	42.5%
**Race**			
African American		25	22%
European American		88	78%
**Stage**			
1a		61	54%
1b		38	34%
1		14	12%
**Smoking Status**			
Never		6	6%
Former		66	58%
Current		41	36%
Missing		0	0%
**Pack-years, mean (SD)**		36.5 (26.9)	
Light smokers (<10 pack-years)		21	19%
Moderate smokers (10-29 pack-years)		33	29%
Heavy smokers (≥30 pack-years)		59	52%
**Vital Status**			
Alive		67	59%
Deceased:			
Lung Cancer Specific		36	32%
Non-Lung Cancer Specific		10	9%

Supplementary Table 8: Coefficients of variances for inter-plate control samples within validation study analysis

	Control 1 %	Control 2 %	Control 3 %	Control 4 %	Control 5 %	Average
**IL-6**	2.9	10.2	12.3	5.7	12.6	8.7
**IL-17A**	7.4	2.1	22.6	37.2	3.9	14.7

Supplementary Table 9: The association of a combined IL-6 & IL-17A prognostic classifier with stage I lung adenocarcinoma survival within the validation study cohort

Classifier: IL-6 & IL-17A	N	%	Univariable			Multivariable*		
			HR	95% C.I.	*P*	HR*	95% C.I.*	*P**
Low for both	55	48.7	1	Reference		1	Reference	
High for one	49	43.4	1.73	0.86-3.49	*0.127*	1.32	0.58-2.99	*0.507*
High for both	9	7.9	2.36	0.77-7.22	*0.133*	2.61	0.67-10.10	*0.165*

P trend: 0.064.
* adjusted for age, gender, stage (1a & 1b), race, smoking (never, former, current) & pack-years.

Supplementary Table 10: Panel of 33 inflammatory markers analyzed

V-PLEX Cytokine Panel Kit 1		V-PLEX Pro-Inflammatory Panel Kit 1	
**Analyte**	**Description**	**Analyte**	**Description**
GM-CSF	Granulocyte-macrophage colony-stimulating factor	IFN-γ	Interferon gamma
IL-12p40	Interleukin-12 subunit p40	IL-10	Interleukin-10
IL-15	Interleukin-15	IL-12 p70	Interleukin-12 subunit p70
IL-16	Interleukin-16	IL-13	Interleukin-13
IL-17A	Interleukin-17A	IL-1β	Interleukin-1 beta
IL-1α	Interleukin-1 alpha	IL-2	Interleukin-2
IL-5	Interleukin-5	IL-4	Interleukin-4
IL-7	Interleukin-7	IL-6	Interleukin-6
TNF-β	Tumor necrosis factor-beta	IL-8	Interleukin-8
VEGF	Vascular endothelial growth factor	TNF-α	Tumor necrosis factor-alpha
**V-PLEX Chemokine Panel Kit 1**		**V-PLEX Vascular Injury Panel**	
**Analyte**	**Description**	**Analyte**	**Description**
Eotaxin	Eotaxin	CRP	C-reactive protein
Eotaxin-3	Eotaxin-3	SAA	Serum amyloid A
IP-10	Interferon gamma-induced protein 10	sICAM-1	Soluble intercellular adhesion molecule-1
MCP-1	Monocyte chemoattractant protein-1	sVCAM-1	Soluble vascular adhesion molecule-1
MCP-4	Monocyte chemoattractant protein-4		
MDC	Macrophage-derived chemokine		
MIP-1α	Macrophage inflammatory protein 1-alpha		
MIP-1β	Macrophage inflammatory protein-1 beta		
TARC	Thymus- and activation-regulated chemokine		

Supplementary Table 11: Coefficients of variance for inter-plate control samples

	Control 1 %	Control 2 %	Average
**CRP**	2.1	0.8	1.5
**Eotaxin**	1.0	2.6	1.8
**Eotaxin-3**	6.0	0.0	3.0
**GM-CSF**	0.9	18.6	9.7
**IFN-γ**	0.3	4.1	2.2
**IL-10**	1.9	4.1	3.0
**IL-12 p70**	0.3	0.2	0.2
**IL-12p40**	5.6	11.3	8.5
**IL-13**	2.5	3.2	2.9
**IL-15**	1.9	1.3	1.6
**IL-16**	6.1	0.4	3.2
**IL-17A**	5.9	14.8	10.4
**IL-1α**	4.1	62.0	33.0
**IL-1β**	7.1	5.3	6.2
**IL-2**	1.1	0.8	1.0
**IL-4**	4.6	5.7	5.2
**IL-5**	12.5	138.2	75.3
**IL-6**	1.1	4.9	3.0
**IL-7**	5.0	10.6	7.8
**IL-8**	7.9	4.7	6.3
**IP-10**	16.6	11.9	14.3
**MCP-1**	5.0	13.7	9.3
**MCP-4**	10.2	11.0	10.6
**MDC**	1.7	6.5	4.1
**MIP-1α**	3.0	5.6	4.3
**MIP-1β**	2.3	3.9	3.1
**SAA**	5.1	0.9	3.0
**sICAM-1**	10.3	3.7	7.0
**sVCAM-1**	4.0	2.6	3.3
**TARC**	9.9	6.1	8.0
**TNF-α**	8.3	6.6	7.4
**TNF-β**	5.1	0.0	2.5
**VEGF**	8.8	6.7	7.8
